# How Does Diurnal and Nocturnal Warming Affect the Freezing Resistance of Antarctic Vascular Plants?

**DOI:** 10.3390/plants12040806

**Published:** 2023-02-10

**Authors:** Dariel López, Carolina Sanhueza, Haroldo Salvo-Garrido, Luisa Bascunan-Godoy, León A. Bravo

**Affiliations:** 1 Departamento de Ciencias Agronómicas y Recursos Naturales, Facultad de Ciencias Agropecuarias y Medioambiente and Center of Plant, Soil Interactions and Natural Resources Biotechnology, Scientific and Technological Bioresource Nucleus, Universidad de La Frontera, Temuco 4811230, Chile; 2 Laboratorio de Fisiología Vegetal, Departamento de Botánica, Facultad de Ciencias Naturales y Oceanográficas, Universidad de Concepción, Concepción 4030000, Chile; 3 Centro de Genómica Nutricional Agroacuícola, Ciencia en Plantas, Temuco 4781158, Chile

**Keywords:** asymmetric warming, climate change, *Colobanthus quitensis*, *Deschampsia antarctica*, freezing resistance, night temperature, supercooling

## Abstract

The Antarctic Peninsula has rapidly warmed up in past decades, and global warming has exhibited an asymmetric trend; therefore, it is interesting to understand whether nocturnal or diurnal warming is the most relevant for plant cold deacclimation. This study aimed to evaluate the effect of diurnal and nocturnal warming on Antarctic vascular plant’s freezing resistance under laboratory conditions. This was studied by measuring the lethal temperature for 50% of tissue (LT_50_), ice nucleation temperature (INT), and freezing point (FP) on *Deschampsia antarctica* and *Colobanthus quitensis* plants. Additionally, soluble carbohydrates content and dehydrin levels were analyzed during nocturnal and diurnal temperatures increase. Nocturnal warming led to a 7 °C increase in the LT_50_ of *D. antarctica* and reduced dehydrin-like peptide expression. Meanwhile, *C. quitensis* warmed plants reduce their LT_50_ to about 3.6 °C. Both species reduce their sucrose content by more than 28% in warming treatments. Therefore, nocturnal warming leads to cold deacclimation in both plant species, while *C. quitensis* plants are also cold-deacclimated upon warm days. This suggests that even when the remaining freezing resistance of both species allows them to tolerate summer freezing events, *C. quitensis* can reach its boundaries of freezing vulnerability in the near future if warming in the Antarctic Peninsula progress.

## 1. Introduction

The increase in the Antarctic vascular plant populations of *Deschampsia antarctica* Desv. and *Colobanthus quitensis* (Kunth) Bartl. observed during the last decades [1,2] has been associated with the regional warming of the Antarctic Peninsula. This has produced longer and warmer growing seasons favoring plant sexual reproduction [3,4,5]. However, Antarctica is among the coldest territories on the planet [6]; therefore, Antarctic plants are constantly dealing with low temperatures, even during growing seasons [7,8].

Several cryoprotective mechanisms have been described to help these plants cope with freezing temperatures, for instance, both vascular plant species living in Antarctica have various morphological xerophytic characteristics, such as small leaf and epidermal cells, thick leaves and cuticle, high stomata density, etc. [9]. A high accumulation of non-structural soluble carbohydrates [10,11,12,13,14] is part of another commonality. In particular, cold-acclimated plants of *D. antarctica* accumulate ice recrystallization inhibition proteins [15,16,17] and dehydrin proteins [18]. However, cold acclimation can easily revert upon exposure to warming, and these cryoprotective mechanisms may get downregulated [16], reducing their effectiveness under the current warming scenario.

Temperature increases above a species-specific threshold could activate plants’ cold deacclimation process, even with short-term exposure [19,20,21]. Cold deacclimation comprises the partial or total loss of previously acquired freezing tolerance [22], this can occur quickly (days to weeks depending on the species) without significant energy cost, leaving the whole plant or some of its tissues vulnerable to subsequent frosts [21,23]. The plants’ frost vulnerability can vary depending on the following: whether it previously reached freezing tolerance, the deacclimation kinetics, and the ability to quickly reacclimate [21]. Cold deacclimation kinetics depend on previous plant cold hardening and the length of the lag phase, during which exposure to warm temperatures does not result in cold deacclimation [23]. The temperature in the Antarctic Peninsula and adjacent islands range between −10 and 15 °C during the growing season [24]. The rise of the minimum night temperatures above the cold deacclimation threshold of *D. antarctica* or *C. quitensis* could threaten their survival. So far, these deacclimation thresholds are unknown for these species.

Field experiments published by Sierra-Almeida et al. [25] reported that *D. antarctica* and *C. quitensis* plants after 2 years of warmer temperatures, generated with Open Top Chambers (OTC), decrease their freezing tolerance in at least one studied site, but this trend was not observed in all studied sites. This variability may be associated with two main issues. First, the OTC warms up only during the day and not at night, in other words, it increases the maximum but does not affect the minimum temperature. This is contrary to model predictions of climate change, which predict a greater increase in minimum night temperatures (nocturnal warming) than the increase in maximum daily temperatures (diurnal warming) [26,27]. This phenomenon is known as asymmetric warming [28] and may increase plant nitrogen mobilization, respiration, and photosynthesis [29,30,31]. Recently, Sanhueza et al. [13] reported that both Antarctic species have differential thermal acclimation of respiration to nocturnal warming.

The other putative explanation is that several environmental variables differ among the studied sites selected by Sierra-Almeida et al. [25] independently of the warming treatment used, such as the mean air temperature, soil composition, nutrient availability, and plant communities. These types of variations are common in field studies, and it is difficult to determine the effect of a specific factor since it is practically impossible to control each factor under natural conditions. For this reason, laboratory studies in growth chambers under controlled conditions are a good option to understand the effect of a single environmental factor (e.g., temperature) on plant physiology.

We propose that nocturnal warming could lead to cold deacclimation of *D. antarctica* and *C. quitensis* rather than diurnal warming. Freezing resistance and cryoprotectants accumulation were evaluated in a short-term laboratory experiment to assess the differential effect of nocturnal vs. diurnal warming on *D. antarctica* and *C. quitensis*.

## 2. Results

### 2.1. Freezing Resistance of D. antarctica and C. quitensis Leaves

**Figure 1:**
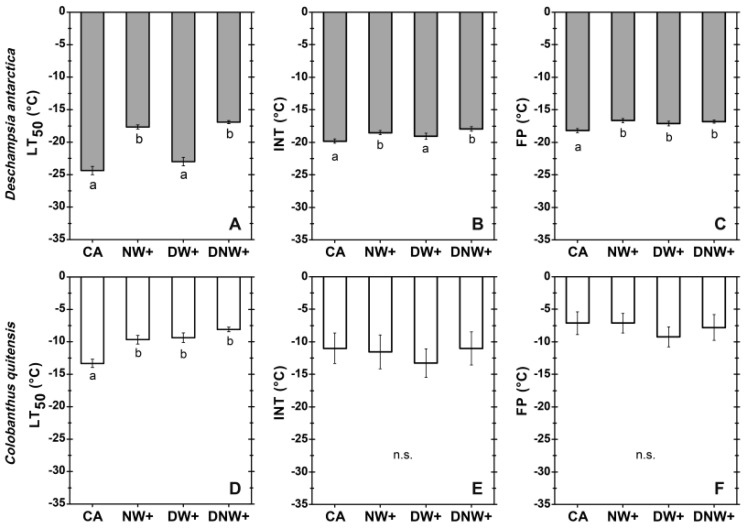
Freezing resistance of *Deschampsia antarctica* (**A**−**C**) and *Colobanthus quitensis* (**D**,**E**). Plants grow in chambers with 18 h/6 h photoperiod and thermoperiods duration. The variables measured were: LT_50_, freezing temperature producing 50% photoinactivation (**A**,**D**); INT, ice nucleation tem−perature (**B**,**E**); and FP, freezing point (**C**,**F**). Values correspond to mean ± SE (*n* = 12 (**A**−**C**) and *n* = 6 (**D**−**F**)), corresponding to experimental treatments with following day/night thermoperiod: 8 °C/0 °C cold−acclimated (CA); 8 °C/6 °C nocturnal warming (NW+); 14 °C/0 °C diurnal warming (DW+); and 14 °C/6 °C diurnal−nocturnal warming (DNW+), with 18 h/6 h photoperiod and thermoperiods duration. Significant differences among treatments are shown as different lower cases (*p* < 0.05). No significant difference (n.s.).

The cold acclimation strategy used (Supplementary Figure S1) allowed cold-acclimated (CA) plants to reach freezing tolerance (LT_50_ values) similar to those reported in the field [25]. Nocturnal warming treatments increased significantly the LT_50_ (*F_3,48_* = 61.10; *p* < 0.001), at least 7 °C higher than control with a small, but still significant, increment (less than 2 °C) of the ice nucleation temperature (INT) (*F_3,48_* = 13.23; *p* < 0.001) and freezing point (FP) (*F_3,48_* = 19.54; *p* < 0.001) in *D. antarctica*. In contrast, diurnal warming treatment (DW+) significantly increased only the FP of this species (Figure 1A–C). Moreover, in *C. quitensis* all warming treatments significantly increased the LT_50_ (*F_3,20_* = 13.24; *p* < 0.001) by at least 3.6 °C with respect to the control treatment, but the INT or FP did not show significant variations among treatments (Figure 1D–F). In addition, the LT_50_ values were lower and statistically different than the corresponding INT in *D. antarctica* plants under CA (*t* = 5.38; *p* < 0.001; *df* = 9) and DW+ (*t* = 5.24; *p* < 0.001; *df* = 9) treatments, with 5.7 °C of thermal difference between these variables; meanwhile, tolerance of apoplastic ice formation was not observed in nocturnal warming (NW+) and diurnal–nocturnal warming (DNW+) treatments given that there was no significant difference between their correspondent LT_50_ and INT. Furthermore, *C. quitensis* leaves did not tolerate apoplastic ice formation since they exhibited statistically similar corresponding values of LT_50_ and INT in any treatment.

### 2.2. Soluble Carbohydrates Content

**Figure 2:**
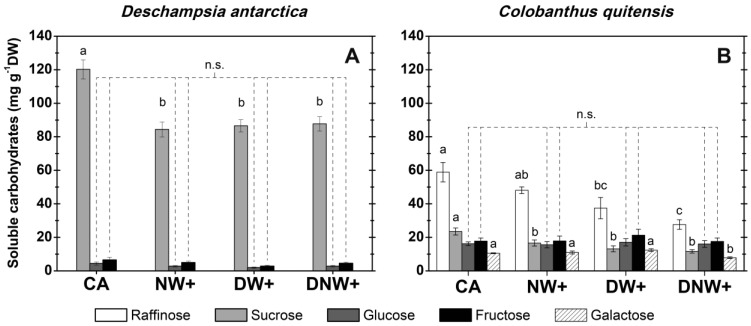
Soluble carbohydrates content in plant leaves of *Deschampsia antarctica* (**A**) and *Colobanthus quitensis* (**B**). Carbohydrates contents are represented by columns: raffinose (white), sucrose (light gray), glucose (dark gray), fructose (black), and galactose (white with diagonal stripes). Values cor−respond to mean ± SE (*n* = 5), corresponding to experimental treatments with the following day/night thermoperiod: 8 °C/0 °C cold−acclimated (CA); 8 °C/6 °C nocturnal warming (NW+); 14 °C/0 °C diurnal warming (DW+) and 14 °C/6 °C diurnal-nocturnal warming (DNW+), with 18 h/6 h photoperiod and thermoperiods duration. Significant differences among treatments are shown as different lower cases (*p* < 0.05). No significant difference (n.s.).

Sucrose was significantly reduced in *D. antarctica* (*F_3,16_ =* 11.22; *p* < 0.001) more than 32.5 mg g^−1^ DW in all warming treatments (Figure 2A). The sucrose content in *D. antarctica* leaves was inversely correlated to their freezing point temperatures (Supplementary Figure S2A). Additionally, in *C. quitensis* sucrose concentration was also significantly reduced in all warming treatments compared to the control (*F_3,16_ =* 9.53; *p* < 0.001) by 6.9 mg g^−1^ DW at least (Figure 2B). Meanwhile, the raffinose content was also significantly reduced in treatments with diurnal warming (*F_3,16_ =* 8.43; *p* = 0.001), with the lowest values measured in plants under DNW+ treatment. The treatment DNW+ presented a significant reduction of galactose content (*F_3,16_ =* 8.52; *p* = 0.001). Furthermore, in *C. quitensis* the raffinose and sucrose variation trends presented an inverse correlation with LT_50_ (Supplementary Figure S2B,C), where the treatment with the highest LT_50_ presented the lowest concentrations of raffinose, sucrose, and galactose. Meanwhile, the fructose and glucose content of both species do not present statistical differences among treatments.

Galactose was not detectable in *D. antarctica* samples. In addition, it was not possible to effectively resolve the raffinose and 1-kestose in *D. antarctica* (Supplementary Figure S3A) or the stachyose and verbascose in *C. quitensis* (Supplementary Figure S3B). However, the heigh of those mixed peaks (in absorbance units) did not vary significantly among treatments (data not show).

### 2.3. Dehydrins Analysis

**Figure 3:**
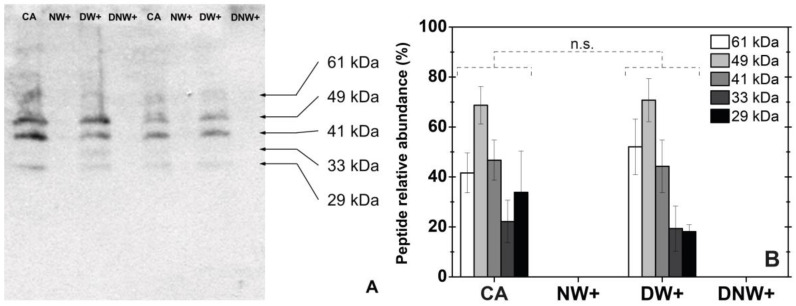
Dehydrin-like peptides from *Deschampsia antarctica* under laboratory conditions. (**A**) Western blot that shows the dehydrin-like peptide accumulation among treatments. (**B**) Relative abundance of dehydrin-like peptides obtained by densitometric analyses of corresponding Western blot. The relative abundance of 5 peptides with different molecular weights was measured. Values represent the mean ± SE (*n* = 5), corresponding to experimental treatments with the following day/night thermoperiod: 8 °C/0 °C cold-acclimated (CA); 8 °C/6 °C nocturnal warming (NW+); 14 °C/0 °C diurnal warming (DW+) and 14 °C/6 °C diurnal-nocturnal warming (DNW+), with 18 h/6 h photoperiod and thermoperiods duration. No significant difference (n.s.).

Five dehydrin-like proteins were detected by the polyclonal antibody against the K segment in *D. antarctica* plants. The detected bands presented apparent molecular weights between 61 and 29 kDa (Figure 3). The increase in nocturnal temperature decreased leaf dehydrin levels to values below the detection limit. However, the increase in diurnal temperature alone did not modify significantly the dehydrin level and profile (Figure 3). No dehydrin-like protein was detected in *C. quitensis*.

## 3. Discussion

Nocturnal warming induced a reduction in the freezing resistance of *D. antarctica* and *C. quitensis* plants in a short-term laboratory experiments. Furthermore, the diurnal warming per se was not able to induce cold deacclimation in *D. antarctica*. This result contrasts with those reported by Sierra-Almeida et al. [25] in field plants, who found that passive warmed plants (daytime only, using open-top chambers) significantly increase their LT_50_ (about 2 °C) in both species in the same study site (Site 2) during the 2015 growing season. This lack of consistency between laboratory and field experiments suggest that other factors, additional to temperature, in the passive warming system could influence the plant response in the field [32]. For instance, the lack of nocturnal warming may have a significant influence on the frequency of freeze/thaw events in the soil and root system, and hence on carbon and nitrogen decomposition [33]. Additionally, in some Alpine plants (e.g., *Prezia carthamoides, Taraxacum officinale,* and *Pozoa coriaceae*), no freezing tolerance difference between OTC passive warming and open space treatments have been reported [34], which show that only diurnal warming is not always enough to induce cold deacclimation.

The increase in night temperature (from 0 to 6 °C), with or without diurnal warming significantly decreased *D. antarctica* freezing tolerance (7 °C), and only slightly reduced (less than 2 °C) the leaf tissue capacity to prevent extracellular ice formation. Growing temperatures above 6 °C, exceed by at least 1 °C the cold deacclimation threshold reported for other Pooideae subfamily members (e.g., *Agrostis stolonifera*, *Lolium perenne*, *Phleum pratense*, *Poa annua,* and *Triticum aestivum*), which is between 3 and 5 °C [19,20,35]. Although current environmental conditions in the Western Antarctic Peninsula do not present regular increases of this magnitude in minimum temperatures, these could occur at least for short-term periods in the near future [26].

On the other hand, *C. quitensis* plants reduced their freezing resistance by more than 3.6 °C at each warming treatment. The fact that *C. quitensis* cold-acclimated plants had similar values of LT_50_ and INT suggests that the cold acclimation conditions used here were not able to induce real freezing tolerance (tolerance of ice crystal formation within the leaf tissue) in this species. Therefore, its freezing tolerance depends on its supercooling abilities, hence their leaves suffered significant damage when apoplastic water freezes. These results are in agreement with those reported by Bravo et al. [36] for *C. quitensis* plants cold-acclimated at 4 °C for 21 days.

In addition, cold-deacclimated plants from this experiment exhibited lower LT_50_ than nonacclimated plants previously reported by Bravo et al. [36] and Gianoli et al. [37]; they were still able to prevent freezing by supercooling to nearly −18 °C and −7 °C for *D. antarctica* and *C. quitensis,* respectively. This is also supported by the small variations in ice nucleation temperature values observed among treatments, being at least 70% and 10% lower for cold-deacclimated plants of *D. antarctica* and *C. quitensis,* respectively, compared to that previously reported by Bravo et al. [36]. The small increase (less than 2 °C) in ice nucleation temperature observed in *D. antarctica* plants under nocturnal warming could be related to an increment of nucleating agents in leaf tissue intercellular spaces [38]. Similarly, the small or null increases in the freezing point of both species (less than 2 °C), suggest that short-term cold deacclimation has little effect on the content of the solutes with colligative properties, as was the case of total soluble sugars reported by Borovik et al. [39] and Sanhueza et al. [13] in wheat and Antarctic vascular plants, respectively.

Despite this, a closer look at the specific soluble carbohydrate’s variation reveals that while glucose and fructose contents remain unchanged at each warming treatment; other carbohydrates, such as sucrose in both species and raffinose and galactose in *C. quitensis*, do decrease their content. Both *D. antarctica* and *C. quitensis* cold-acclimated plants commonly accumulate large amounts of sucrose [10,40,41], which has a wide cellular distribution and could have an important role, not only by depressing the freezing point colligatively [42,43] but also by stabilizing proteins and cell membranes [44,45].

Sucrose was the most abundant soluble carbohydrate in *D. antarctica*, and a 28% drop of sucrose content in DW+ plants does not affect freezing tolerance in this species. However, at least part of this sucrose content reduction seems to be reflected in the colligative properties of the apoplastic fluid, with a modest increase in its freezing point. In contrast, sucrose seems to have a more relevant role in freezing resistance as a cellular protector in *C. quitensis*, since a 30% drop in sucrose content could be related to a reduction in freezing resistance of its leaf tissue when growth temperature increases. In addition, sucrose did not colligatively increase the freezing point in *C. quitensis*.

Another *C. quitensis* soluble carbohydrate with high variation among treatments is raffinose, which is produced in the cytoplasm but accumulates mainly in the vacuole and chloroplast stroma, where it is believed to serve as a membrane integrity protector [46,47]. Raffinose is also associated with the protection and stability of PSII [48]. Therefore, the decrease in leaf raffinose contents could contribute to the increase in LT_50_ in warmed plants of *C quitensis* in our experiment, as was reported in other Eudicots, including wild blueberry [49] or sugar beet [50] and also boreal conifers [51]. This could be particularly important because our LT_50_ method is essentially based on PSII stability.

We were unable to separate and quantify oligofructans previously reported for this plants species [11,12], such as raffinose and 1-kestose (*D. antarctica*), and stachyose and verbascose (*C. quitensis*). However, the added unresolved peaks did not show apparent differences among deacclimation treatments. This suggests that these sugars do not respond to short-term warming.

The decrease of accumulated soluble carbohydrates could be related to their allocation to plant vegetative growth and the increase in the respiration rate activated by warmer temperatures [21,29,52]. Previous results in Antarctic vascular plants seems to support this hypothesis since Sanhueza et al. [13] reported that *D. antarctica* and *C. quitensis* under nocturnal warming experimental condition reduced their ratio between respiration and carbon assimilation, favored mainly by the increase of CO_2_ assimilation in *D. antarctica* and acclimation of respiration in *C. quitensis*. Night-warmed *D. antarctica* also increases its dark respiration during the day [13]. Meanwhile, Sáez et al. [53] observed that *C. quitensis* plants under passive warming treatment (similar to DW+) reduced their leaf mass area and leaf density and promoted plant growth, net CO_2_ assimilation rate, and dark respiration. This suggests that the accumulated carbohydrates and the newly assimilated CO_2_ were used in other biological processes, such as vegetative and reproductive growth.

Similar levels of dehydrin-like proteins in CA and DW+ plants of *D. antarctica*, as well as their absence in the treatments including nocturnal warming, suggest an important role in the freezing tolerance in this species and further support the idea that nocturnal more than diurnal warming favors the cold deacclimation response in the Antarctic grass. Dehydrins are associated with cell membrane stability, preventing membrane hexagonal-phase transition under freezing temperature as a result of lipids interaction in a medium with less polarity, and the consequent electrolytes loss and other essential components of the cytoplasm [54]. The observed decrease in dehydrin-like protein levels during cold deacclimation has also been recorded in winter wheat and grasses, such as *Poa annua*, *Agrostis stolonifera,* and *Cynodon* spp. [19,39,55,56]. Therefore, given that diurnal warming *per se* does not affect the relative abundance of the dehydrin-like proteins detected in *D. antarctica*, it could be inferred that DW+ has little effect on the cold deacclimation induction in this species, at least in the current experimental condition. In the same line of thought, it could be expected that nocturnal warming with minimum night temperatures above the deacclimation threshold, downregulated the transcription factors required for dehydrin expression as it happens in *Hordeum vulgare* and *Rhododendron anthopogon* plants [57,58], as well as other proteins related to freezing tolerance, such as apoplastic ice recrystallization inhibition proteins (IRIP) [15,59,60]. A prominent candidate to regulate the expression of these genes is CBF transcription factor, which conforms a regulon that controls the expression of genes necessary for cold acclimation and freezing tolerance [61,62,63,64]. Two CBF genes have been described and proved to be functional in *D. antarctica* [65]. The analyses of the regulon CBF transcripts, comparing diurnal and nocturnal warming, are under way.

It is intriguing the absence of dehydrin antibody recognition in *C. quitensis*, even in cold-acclimated plants. It seems that the antibody had no cross reactivity with this Caryophyllaceae, even when it is capable to detect dehydrins in other Eudicots families, such as Malvaceae, Solanaceae, Brassicaceae, Fabaceae, and Cucurbitaceae [66].

The results obtained in this research are relevant to predict future impacts of regional warming in Antarctica concerning plant freezing vulnerability, since our results suggest that even the observed reduction in freezing tolerance/avoidance due to cold deacclimation is not enough to jeopardize plant survival. These plant species could withstand freezing events of around −17 °C for *D. antarctica* and up to −8 °C for *C. quitensis*, even though field plants could respond slightly differently to the same stimulus. These would allow both plant species to cold-deacclimate within a safety margin, being not yet vulnerable to the lowest freezing event reported for Antarctic summer months (−6.7 °C) near H. Arctowski Station by Kejna [67] and Plenzler et al. [68]. However, if *C. quitensis* plants would cold-deacclimate earlier in the growing season, they will approach dangerously to the vulnerability range of temperature because temperatures can reach as low as −16 °C between October and November [67,68].

## 4. Materials and Methods

### 4.1. Plant Material, Growth Conditions, and Warming Treatments

*Deschampsia antarctica* Desv. and *Colobanthus quitensis* Kunth Bartl. plants were collected in February 2018 near Henryk Arctowski Polish Scientific Station, King George Island, Maritime Antarctic (62°09′41″ S–58°28′10″ W). Plants were reproduced vegetatively and grown as described by Bravo et al. [36] in 400 mL pots with soil mixture 3:2:1 (vegetal soil:vermiculite:peat) in a controlled climate room at 13 °C constant temperature with 18 h light and 6 h dark photoperiod, which coincides with their habitat photoperiod in November according to NOAA ‘Sunrise/Sunset Calculator’, when snow cover is usually melted at the beginning the growth and reproduction period in their natural habitat.

These plants were cold acclimated at 10° C/2 °C (max/min) of 18 h/6 h day/night thermoperiod. Plants were maintained for 14 days under these conditions, then the diurnal temperature was reduced to 8 °C and the nocturnal temperature to −2 °C for 7 days to induce further cold acclimation, and subsequently the nocturnal temperature was increased to 0 °C for 7 more days. This condition maintains cold acclimation while it is close to Antarctic Spring temperatures (28 days in total). Then, groups of pots were transferred to 4 thermoperiods treatments that were established with the following temperature of culture: (CA) maintained at 8 °C/0 °C day/night, it is the control where plants remain in cold acclimation condition; (NW+) only nocturnal warming at 8 °C/6 °C day/night; (DW+) only diurnal warming 14 °C/0 °C day/night; and (DNW+), both diurnal and nocturnal warming at 14 °C/6 °C day/night. In all conditions, the photoperiod was maintained the same, the minimum temperature lasted 6 h within the night period, and the maximum temperature was maintained for 10 h of the day period, leaving the remaining 8 h of the day for the gradual increase and decrease of temperature. The plants remained in these conditions for 14 days, and the treatment’s effect was evaluated at the end of this period.

### 4.2. Freezing Tolerance (LT_50_)

The freezing tolerance was determined in *D. antarctica* (*n* = 10) and *C. quitensis* (*n* = 6) plants as the temperature at which leaf tissue reached 50% of photoinactivation (PhI), interpreted as the lethal temperature for 50% of tissue (LT_50_). This parameter was evaluated on excised leaves by applying a controlled temperature decent using a Peltier-Thermoelectric Cold Plate Cooler (CP-121, TE-Technohlogy, Inc., Traverse City, MI, USA), regulated by a bidirectional temperature controller, programmed at desired temperature through software supplied by the manufacturer (TE-Technology, Inc., Traverse City, MI, USA). The leaves were spread on slightly moistened filter paper on the Peltier modules surface and covered with a transparent glass to avoid tissue dehydration and to obtain close thermal contact of leaves to the cold plate. Maximum photochemical efficiency (*Fv*/*Fm_initial_*) was determined after darkening the plant material for 30 min at 13 °C (control temperature) with the IMAGING-PAM M-Series MAXI Version fluorimeter (Walz, Effeltrich, Germany). Then, the temperature was lowered from 13 to 0 °C within 30 min and then at different target temperatures, from −6 to −35 °C (one target temperature for each Peltier); once the target temperature was reached, it was maintained for 90 min. Subsequently, the temperature was raised again to 0 °C, where it was kept for 30 min (tissue thawing period), and then from 0 to 13 °C, where it remained for 9 h (recovery period). Leaves were in darken all the time. Once this period had elapsed, *Fv*/*Fm* has measured again (*Fv/Fm_final_*) (Supplementary Figure S4). The minimum *Fv/Fm* after a deathly freezing event was obtained by submerging the leaves in liquid nitrogen for 90 min, then putting them in a 0 °C environment for 30 min; after that, the leaves were organized on a moistened filter paper and kept on a covered Peltier modules surface at 13 °C for 9 h (*Fv/Fm_death_*) in darkness. To calculate the percentage of photoinactivation (PhI) was used the following formula modified from Larcher [69]: %PhI = (*Fv/Fm_initial_* − *Fv/Fm_final_*)/(*Fv/Fm_initial_* − *Fv/Fm_death_*) × 100.

### 4.3. Thermal Analysis

This analysis was used to determine the ice nucleation temperature (INT) and the freezing point (FP) of *D. antarctica* (*n* = 10) and *C. quitensis* (*n* = 6) leaves. It was carried out based on the methodology described by Arnold et al. [70] with modifications. The first fully expanded leaves of the sprout were used. These leaves were placed on the Peltier-Thermoelectric Cold Plate Cooler surface (CP-121, TE-Technology, Inc., Traverse City, MI, USA) covered with parafilm to decrease the thermal conductivity of the metal plate. A thermocouple (Gauge 30, copper-constantan, type K thermocouples; Cole Palmer Instruments, Vernon Hills, IL, USA) was placed on each leaf to monitor the temperature. Later the system was covered with a sheet of foam rubber to insulate it thermally. The Peltier was controlled using a program supplied by PITEC (Puig Ingeniería y Tecnología S.A., Santiago, Chile), regulating the temperature drop from 0 to −24 °C at 3 °C h^−1^ rate. The leaf temperature was monitored with a personal data acquisition module (DAQ, USB-2408 Series) controlled by DAQami™ software, both supplied by the manufacturer (Measurement Computing Corp, Norton, MA, USA). The leaf INT and FP were determined based on the exotherms produced by the heat released during the process of water freezing in the apoplast, including the water from the symplast driven outwards by water potential difference caused by apoplastic ice formation [71]. The low temperature value at the exotherm start corresponds to ice INT, and the maximum point reached by the exotherm corresponds to FP.

### 4.4. Carbohydrate Analyses

Soluble carbohydrates were obtained from 15 mg of lyophilized leaf tissue from 5 plants of each species. Total soluble sugars (TSS) content was extracted three times with methanol:chloroform:water (12:5:3, *v*/*v*/*v*) solution and separated from nonpolar pigments and lipids by adding ⅓ and ¼ final volume of chloroform and water, respectively, according to Dickson [72]. High thin-layer chromatography was carried out to determine the amount of fructose, glucose, sucrose, and raffinose following the protocol proposed by Oberlerchner et al. [73] with modifications. In summary, prewashed in methanol:water (6:1, *v*/*v*) silica-gel glass plates 60 F_254_ (20 × 10 cm, Merck, Darmstadt, Germany) were impregned with sodium dihydrogen phosphate-disodium hydrogen phosphate buffer (pH 6.8, 0.2 M) by immersion. Samples and standards were sprayed as bands of 8 mm using an ATS4 automated applicator (CAMAG, Muttenz, Switzerland). The distance between bands was 2 mm with bands starting at 8 mm from the bottom of the plate. Different known amounts of fructose, glucose, galactose, sucrose, raffinose, and stachyose standards were sprayed on 6 lines in all plates. All samples were sprayed twice, once with 2 µL volume for oligosaccharides and the other with 10 µL, for monosaccharides quantifications. Development was carried out in three sequential steps in a semiautomated chamber (CAMAG Twin Through Glass chamber 20 × 20 cm). First and second developments used as solvent acetonitrile:1-pentanol:water (4:1:1, *v*/*v*/*v*), while the third changed to acetonitrile:1-butanol:water (4:1:1, *v*/*v*/*v*). Every development was set: predrying 30 s, no saturation, 10 min activation with MgCl_2_ solution, and 70 mm migration distance each run. To allow detection, sugar derivatives were generated by dipping the plates 1 s in a solution containing aniline 1% (*v*/*v*), diphenylamine 1% (*w*/*v*), orthophosphoric acid 9% (*v*/*v*), and methanol:water (9:1, *v*/*v*). Plates were then heated at 130 °C for 5 min and scanned (TLC scanner 3, CAMAG, Muttenz, Switzerland) at 520 nm. The quantification was carried out via peak high and a multilevel calibration with linear regressions, using the Software WinCATS 1.4.4.6337 (CAMAG, Muttenz, Switzerland).

### 4.5. Immunoblot Analyses of Dehydrin-Like Peptides

Extraction of the thermostable fraction of proteins was performed from the leaf tissue of 5 plants of each species, based on the method proposed by Oliveira et al. [74] with modifications. In summary, liquid nitrogen frozen plant material was lyophilized and pulverized in mortars. A total of 50 mg dry leaf tissue from each sample was added 1 mL extraction buffer (20 mM TES, 0.5 M NaCl, pH 8.0). The mixture was vigorously shaken for 1 min and then centrifuged at 15,000× *g* at 4 °C for 10 min. Subsequently, the supernatant was boiled at 100 °C for 10 min then chilled on ice and centrifuged. Soluble proteins were quantified by the Bradford assay [75]. Then, the proteins were precipitated in TCA 15% (*w*/*v*), and after centrifuge at 15,000× *g* 4 °C for 15 min, the pellet was washed twice with a solution of methanol and 0.1 M ammonium acetate and dried in a desiccator. Next, the pellet was resuspended in Laemli buffer and 20 μg of proteins from each sample were separated in duplicate according to their molecular weight using SDS-polyacrylamide gels electrophoresis, composed of a stacking gel 4% (*w*/*v*) and a separating gel 12% (*w*/*v*). One gel was stained with colloidal Coomassie brilliant blue G-250 [76]. Meanwhile, the other was transferred to a PVDF membrane by wet transfer (1 h at 110 V). Dehydrin immunolocalization was carried out using polyclonal antibodies with reactivity against the Lysine-rich peptide (K segment) of dehydrins at the manufacturer’s recommended dilution 1:4000 [77], and a secondary HRP-conjugated antirabbit IgG (Bio-Rad, Hercules, CA, USA) in a dilution 1:10,000. Protein detection was carried out by ECL chemiluminescence (Pierce, Rockford, IL, USA) on X-ray film (Fuji, Tokyo, Japan). The densitometric measurements of the bands corresponding to each protein were performed with the TotalLabQuant software. The results were expressed as a percentage of the maximum level determined.

### 4.6. Statistical Analyses

The effect of diurnal and nocturnal warming on LT_50_, INT, FP, and carbohydrates content was assessed using one-way ANOVA tests. The differences among treatments in the ANOVA were assessed by Tukey-HSD tests. Also, the difference between the temperatures at which LT_50_ and INT occur and the dehydrins levels were assessed with the Student’s *t*-test. Finally, the relations between significantly variable carbohydrates and FP for *D. antarctica* or LT_50_ for *C. quitensis* were assessed with the Pearson correlation test. The analyses were performed with the software Statistica 8.0 (Stat Soft Inc. Tulsa, OK, USA).

## 5. Conclusions

The evidence obtained in the present study partially supports our hypothesis, since an increase in minimum night temperature is key in the cold deacclimation process in both species. In contrast, a short-term increase in maximum diurnal temperature only induced a significant cold deaclimation in *C. quitensis*. Furthermore, the remaining freezing resistance of *D. antarctica* and *C. quitensis* allows them to tolerate the magnitude of possible freezing events that occur during the summer months in their habitat, but *C. quitensis* could be more vulnerable to freezing if warming in the Antarctic Peninsula continues. However, more studies are necessary to understand the mechanisms that lead to the cold deacclimation of these species as well as to better understand the consequences that regional warming will have on their field populations. In this sense, it would be interesting to evaluate the effect of night warming under field experimental conditions with the development of new active warming systems that allow controlling the minimum temperatures.

## Data Availability

Not applicable.

## Supplementary Materials

The following supporting information can be downloaded at: https://www.mdpi.com/article/10.3390/plants12040806/s1, Figure S1: Cold acclimation strategy and treatment design. Graphic description of the cold acclimation process used in the experiment (upper part of the figure). The different warming treatments used for 14 days before assessing the experiment: control cold-acclimated plants (CA), nocturnal warming (NW+), diurnal warming (DW+), and diurnal-nocturnal warming (DNW+). The black and white bars above the x axis represent the dark (night) 6h and light (day) 18h daily periods respectively. The red arrow highlights the temperature increments used in each treatment (bottom part of the figure); Figure S2. Significant correlation between soluble carbohydrates content and freezing resistance variables. *Deschampsia antarctica*: sucrose content vs freezing point (A). *Colobanthus quitensis*: raffinose content vs LT_50_ (B), and sucrose content vs LT_50_ (C). Each graph represents 5 biological replicates in the 4 treatments (CA, NW+, DW+, DNW+), with *p < 0.001* significant. The 95% confident intervals are represented between dash lines; Figure S3. Soluble carbohydrates profiles. Densitometric histogram of the absorbance vs retention factor (Rf) of soluble carbohydrates recorded from *Deschampsia antarctica* (A) and *Colobanthus quitensis* (B) in silica plates pretreated with sodium phosphate buffer, after two development with acetonitrile:1-pentanol:water (4:1:1) and one with acetonitrile:1-butanol:water (4:1:1). Labeled in black are the peaks that were possible to resolve and quantify, meanwhile in red are labeled the peaks mixed that was not possible discriminate between their putative components; Figure S4. Photosystem II damage after freezing events. Visual representation of *Fv/Fm_final_* values of *Deschampsia antarctica* group of leaves, after different freezing events and their control at 13 °C. Experimental treatments represent the following day/night thermoperiod: 8 °C/0 °C cold-acclimated (CA); 8 °C/6 °C nocturnal warming (NW+); 14 °C/0 °C diurnal warming (DW+) and 14 °C/6 °C diurnal and nocturnal warming (DNW+). In the picture bottom is presented the percentual scale of *Fv/Fm* values.
