# Supplementary material for: How Does Diurnal and Nocturnal Warming Affect the Freezing Resistance of Antarctic Vascular Plants?
Source: Plants (Basel). 2023 Feb 10;12(4):806. doi: 10.3390/plants12040806 (PMC9966323; doi:10.3390/plants12040806)
Supplement: Supplementary file 1 [file plants-12-00806-s001.zip › supplementary materials.pdf]

Figure S1.

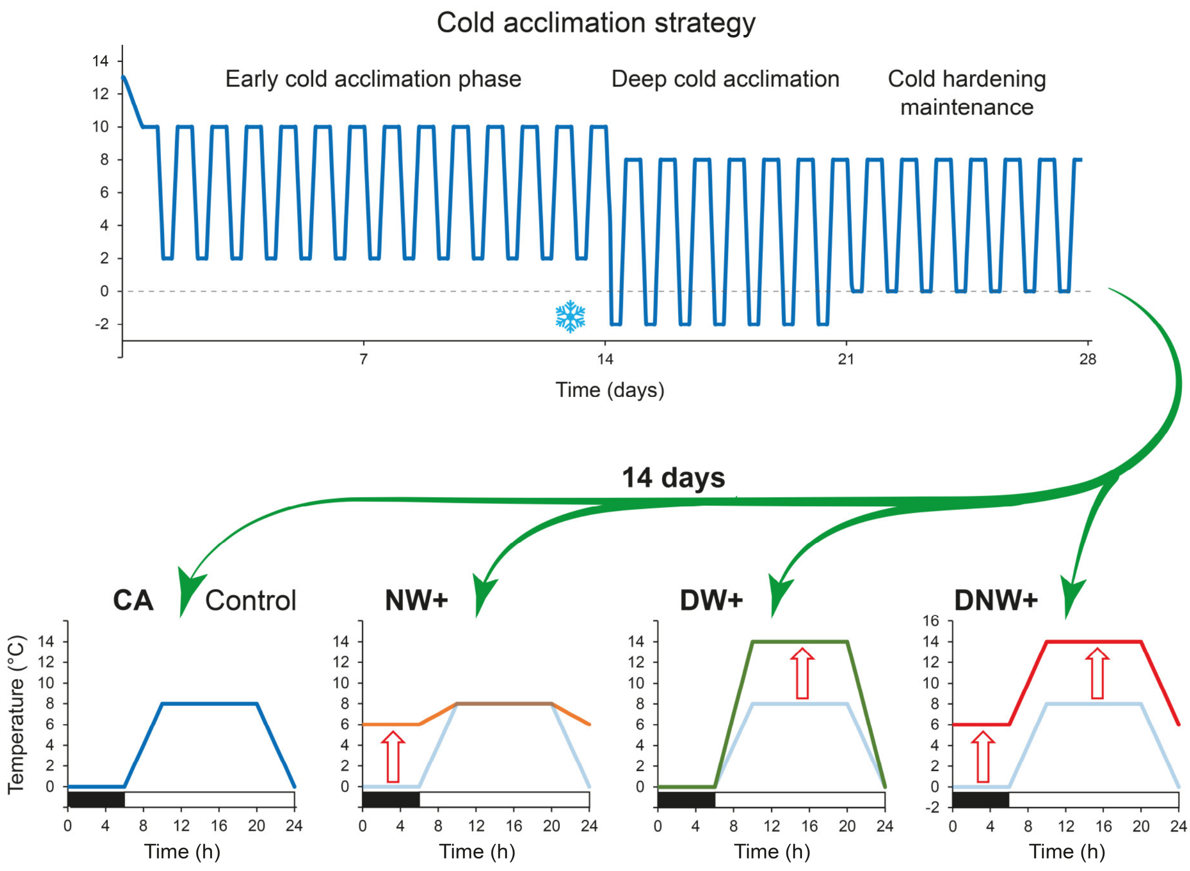

Figure S1. Cold acclimation/deacclimation strategy and treatment design. Graphic description of the two different experimental phases. First cold acclimation (cold-hardening) it is showed in the upper panel and takes 28 days. The Second phase was cold-the deacclimation process (or warming treatments) which takes 14 days (lower panel). The different warming treatments were as follows: control cold-acclimated plants (CA), nocturnal warming (NW+), diurnal warming (DW+), and diurnal-nocturnal warming (DNW+). The black and white bars above the x-axis represent the dark (night) 6h and light (day) 18h daily periods respectively. The red arrow highlights the temperature increments used in each treatment (bottom part of the figure).

Figure S2

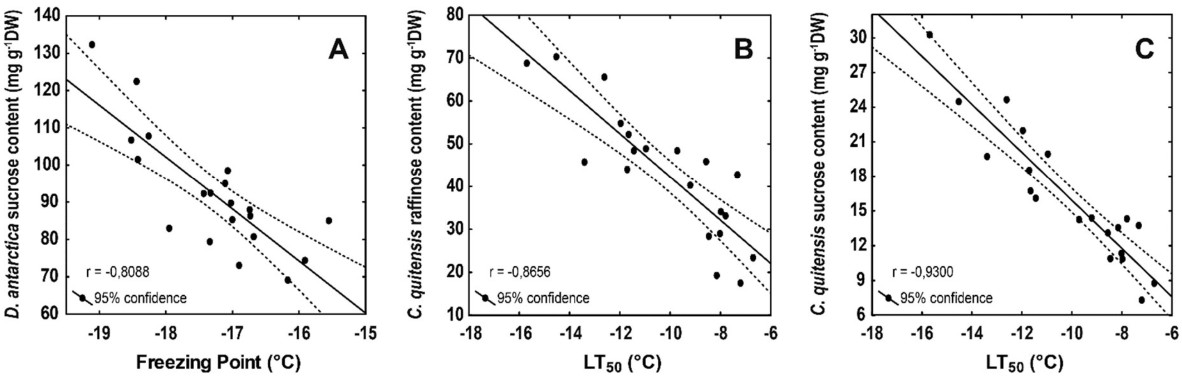

Figure S2. Significant correlation between soluble carbohydrates content and freezing resistance variables. *Deschampsia antarctica*: sucrose content vs freezing point (A). *Colobanthus quitensis*: raffinose content vs LT_{50} (B), and sucrose content vs LT_{50} (C). Each graph represents 5 biological replicates in the 4 treatments (CA, NW+, DW+, DNW+), with a significant $p < 0.001$. The 95% confidence intervals are represented between dash lines.

Figure S3

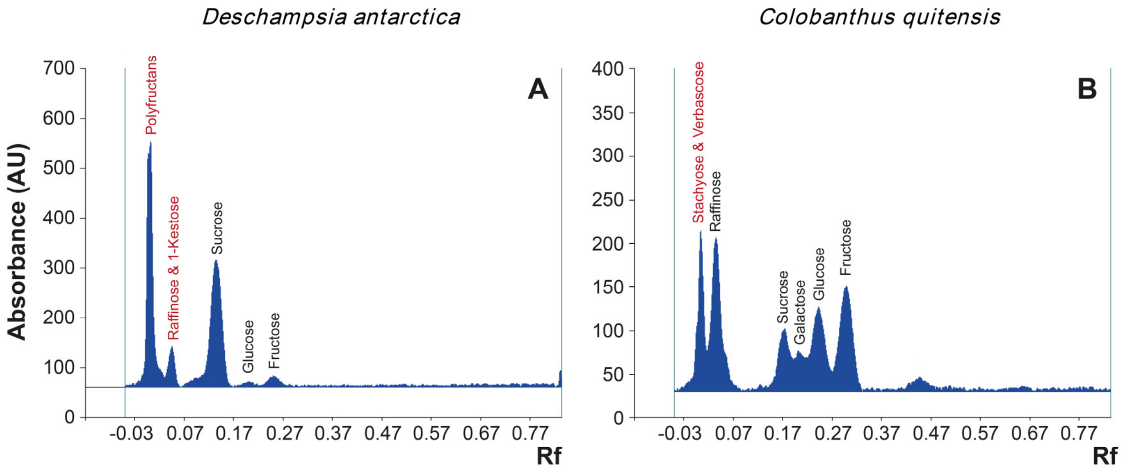

Figure S3. Soluble carbohydrates profiles. Densitometric histogram of the absorbance vs retention factor (Rf) of soluble carbohydrates recorded from *Deschampsia antarctica* (A) and *Colobanthus quitensis* (B) in silica plates pretreated with sodium phosphate buffer, after two developments with acetonitrile:1-pentanol:water (4:1:1) and one with acetonitrile:1-butanol:water (4:1:1). Labeled in black are the peaks that were possible to resolve and quantify, meanwhile in red are labeled the mixed peaks that was not possible discriminate between their putative components.

Figure S4

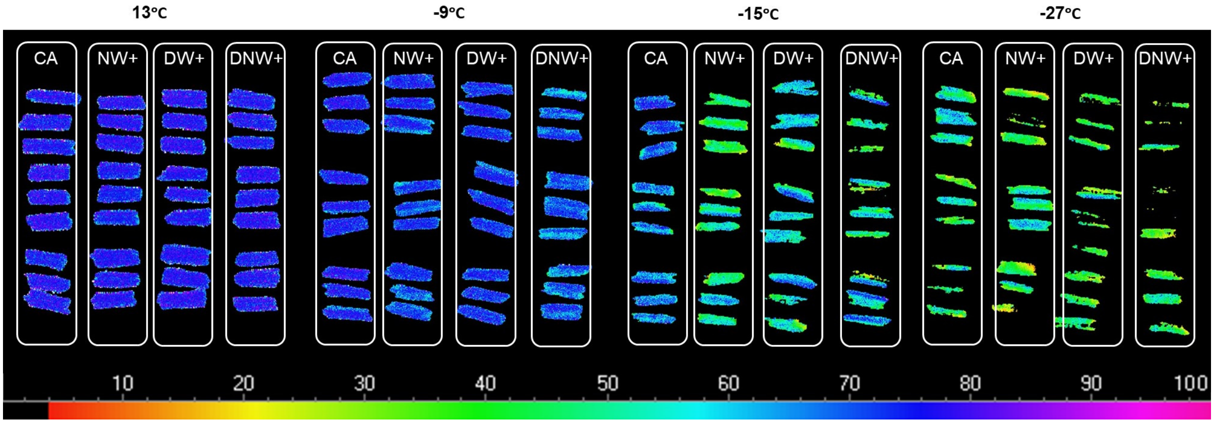

Figure S4. Photosystem II damage after freezing events. Visual representation of *Deschampsia antarctica* leaves *Fv*/*Fm* post freezing images, after different target freezing temperatures (-9, -15, -27 °C) and their nonfrozen control. All *Fv*/*Fm* measurements were performed at 13°C. Experimental treatments represent the following day/night thermoperiod: 8°C/0°C cold-acclimated (CA); 8°C/6°C nocturnal warming (NW+); 14°C/0°C diurnal warming (DW+) and 14°C/6°C diurnal and nocturnal warming (DNW+). The bottom scale represents the *Fv*/*Fm* values as percentage.
